# Persistent Median Artery Prevalence: A Cadaveric Study

**DOI:** 10.7759/cureus.101539

**Published:** 2026-01-14

**Authors:** Paul Tran, Dallas Bennett, Elizabeth A Eversole, Areeba Al-Sharfeen, Victoria Nguyen, Aleah C Frison, Camilla Arguedas, Chakravarthy Sadacharan, Adi Pinkas

**Affiliations:** 1 Anatomy, Tilman J. Fertitta Family College of Medicine, Houston, USA; 2 Pharmacy, University of Houston College of Pharmacy, Houston, USA; 3 Anatomy, Baylor College of Medicine, Houston, USA

**Keywords:** bifid median nerve, carpal tunnel, median nerve, mn, persistent median artery, pma

## Abstract

Introduction: The persistent median artery (PMA) is a blood vessel present during early embryonic development via morphogenesis. Due to its proximity to the median nerve (MN), the PMA is often associated with pain, especially related to carpal tunnel syndrome, anterior interosseous nerve compression, and hand motor dysfunction. As previous literature has reported a wide spectrum of data on prevalence, this cadaveric study aims to not only assess the prevalence of PMA but also collect vessel data in relation to the median nerve.

Methods: Cadaveric human forearms (N = 102) were dissected and identified for the prevalence and orientation of PMA. Measurement of vessel lumen thickness was also performed. Data points were then compared to previous literature to assess for any remarkable differences in findings.

Results: The prevalence from this dissection study was approximately 12.7% (13/102). Orientation of the PMA in relation to the median nerve was noted to be found in various configurations, including anterior-piercing (61.5%, 8/13), laterally (30.8%, 4/13), or medially (7.7%, 1/13). Unilateral PMA distribution (9/13) was found to be greater than bilateral distribution (4/13). The average measured diameter of the PMA was found to be 0.45 ± 0.15 mm.

Conclusion: Findings from the present study are moderately consistent with previous literature, as the anterior-piercing type is most reported. Prevalence is also within range compared to previous studies. Assessing the prevalence, location, and orientation of the PMA in reference to the proximity of the carpal tunnel is crucial, as this vessel is linked to carpal tunnel compression and postoperative complications.

## Introduction

The persistent median artery (PMA) is a blood vessel present during early embryonic development. Although the PMA usually dissipates during fetal life apoptosis, it sometimes persists after birth due to failure of complete cellular regression [1]. Previous literature has shown that the PMA provides blood supply to intrinsic hand muscles as it transcends the distal forearm and eventually through the carpal tunnel [1,2].

When present, the PMA is known to contribute to the accessory blood supply and partial formation of the superficial palmar arch [2]. As this artery extends into the palmar side of the hand, it also has clinical implications in relation to carpal tunnel. As this structure is commonly involved in various forearm and hand procedures, the location and existence of the PMA should be considered. As the PMA is commonly known to develop in close proximity to the median nerve (MN), it is often associated with pain related to carpal tunnel pressure, anterior interosseous nerve compression, and blood supply problems [1,3-5]. Although there is a high variation in the prevalence of the PMA reported in previous literature, recent statistics in 2021 have shown a pooled PMA prevalence of 8.6% in the general adult population [6]. Although previous studies have shown important relationships between the PMA and other important intrinsic forearm structures, there is much variation in reported data regarding PMA prevalence and morphology.

This study aims not only to assess the prevalence of the PMA via a cadaveric study but also to collect parametric data that may have important implications related to common upper extremity pathology.

## Materials and methods

Cadaveric human forearms (N = 102) from 26 male and 25 female donors were dissected and identified at the University of Houston Tilman J. Fertitta Family College of Medicine and Baylor College of Medicine for both the prevalence and patterns of the PMA. Measurement of vessel diameter thickness was also taken via digital caliper. Cadaveric donors were stored at appropriate temperatures within formaldehyde submersion and storage compartments to ensure ideal preservation conditions. Parametric data were then collected and entered into a spreadsheet to be compared to previous literature and assessed for any significant contrast in findings.

Inclusion and exclusion criteria

Cadaveric forearms that were of atraumatic appearance with intact vessels were included in the study. Forearms with muscle or vessel abnormalities or under improper preservative conditions were excluded from the study.

Cadaveric forearms were grossly dissected, and the appropriate forearm muscles (including pronator teres, flexor carpi radialis, palmaris longus, and flexor carpi ulnaris) were then retracted. If the PMA was identified, exposure of the surrounding intrinsic structures was then analyzed for orientation of the PMA. The PMA was then traced retrogradely from the proximal entry point of the carpal tunnel toward the cubital fossa. The arterial vessel was additionally verified by manual compression, and the diameter measurements were taken.

## Results

**Figure 1:**
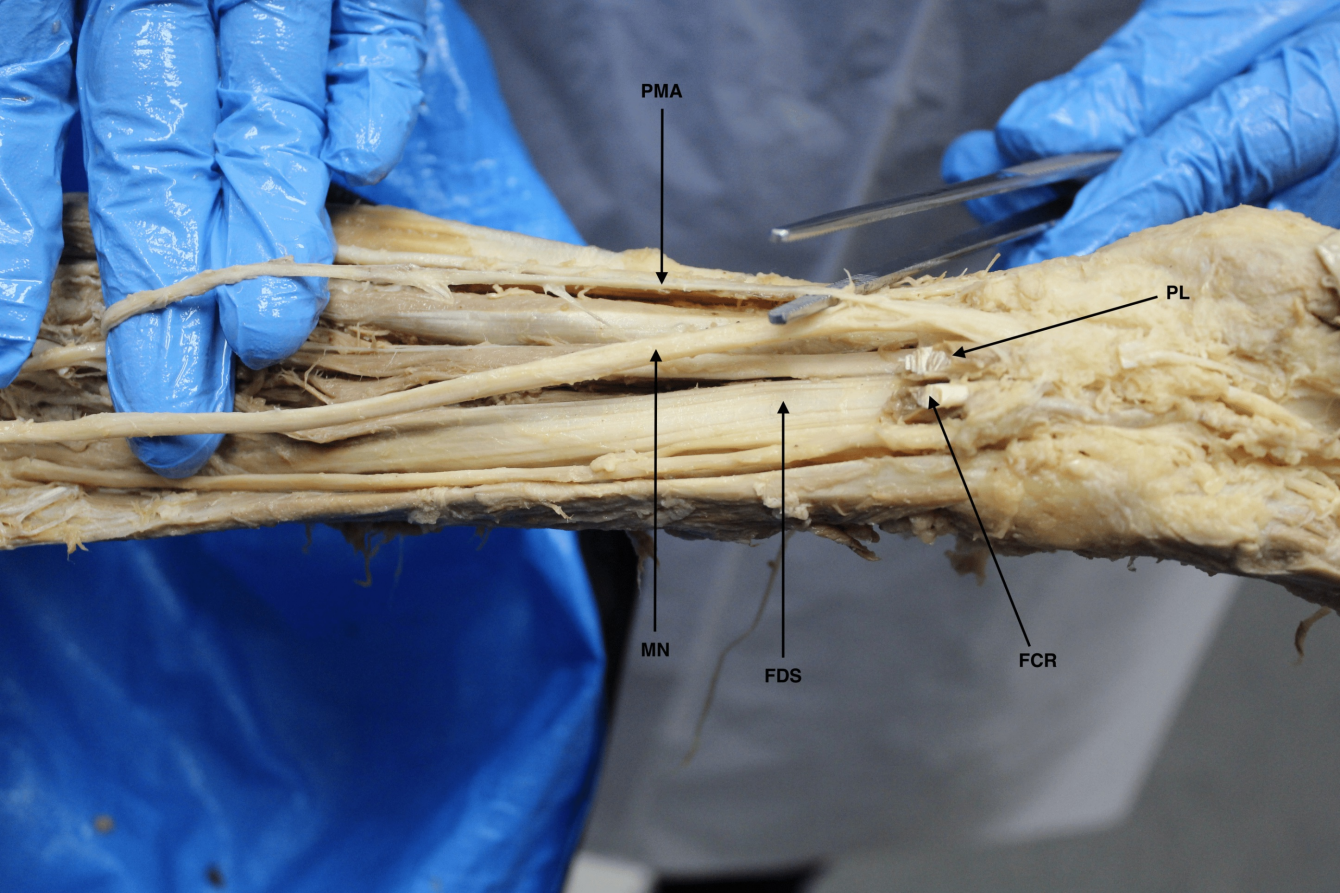
Cadaveric forearm showcasing a PMA incident crossing anteriorly to the MN before piercing the MN inward, splitting the terminal ends, and entering the carpal tunnel PMA: persistent median artery, MN: median nerve, FDS: flexor digitorum superficialis, PL: palmaris longus, FCR: flexor carpi radialis

The PMA was found in 13 cadaveric forearms (12.7%) from this dissection study. The orientation of the PMA in relation to the median nerve was found in various configurations, including anterior-piercing in eight forearms (61.5%) (Figure 1), laterally in four forearms (30.8%), and medially in one forearm of a unilateral-sided donor (7.7%). Furthermore, unilateral PMA distribution was found to be greater in comparison to bilateral distribution. A summary of the data findings can be found in Table 1. The average measured diameter of the PMA was found to be approximately 0.45 ± 0.15 mm.

**Table 1:** Parameter, prevalence, and count data from the cadaveric dissections of PMA PMA: persistent median artery

Parameter	Prevalence (%)	Count (n/N)
Overall prevalence	12.7%	13/102
Orientation of PMA		
Anterior-piercing	61.5%	8/13
Lateral	30.8%	4/13
Medial	7.7%	1/13
Distribution		
Unilateral	69.2%	9/13
Bilateral	30.8%	4/13

## Discussion

Findings from the present study are moderately consistent with previous literature, as the anterior-piercing orientation is the most prominent reported pattern type, which closely resembles palmar type formation [4,5,7]. A table of previous studies with reported sample size and prevalence percentage has been constructed in chronological order (Table 2). PMA prevalence is also comparable to that of previous studies. However, the measured average diameter is noted to be smaller compared to various ultrasound and Doppler studies screening asymptomatic volunteers for carpal tunnel with measurements ranging from 0.5 mm and upward [5,8]. Findings from the study by Chen et al. demonstrated that patients with PMA diameters ranging from 1.0 to 1.5 mm were asymptomatic, whereas those with PMA diameters of 3.0 mm and upward had symptomatic carpal tunnel syndrome [7]. In a study conducted by Gassner et al., two patients with symptomatic swelling of the hands had to undergo release of the transverse carpal ligament [5].

**Table 2:** Chronological chart highlighting various studies that have been conducted in relation to PMA prevalence PMA: persistent median artery

Study	Year	Sample size (number)	Prevalence (%)	Type of study
Weathersby [9]	1954	218	9.6	Cadaveric
Libersa et al. [10]	1982	100	16.0	Cadaveric, radiological
Henneberg and George [11]	1995	516	31.6	Cadaveric
Olave et al. [12]	1997	102	22.6	Cadaveric
Henry et al. [13]	2005	200	19.0	Cadaveric
Nayak et al. [14]	2010	84	15.5	Cadaveric
Stimpson and Gupta [15]	2012	100	19.0	Radiological
Patnaik and Paul [16]	2016	100	6.0	Cadaveric
Yildiz et al. [17]	2019	121	52.1	Cadaveric
Sophia et al. [18]	2021	42	57.1	Cadaveric
Current study	2024	102	12.7	Cadaveric

Previous literature conducting prevalence studies has reported similar characteristics as described within the study findings. In many studies, the anterior-piercing type seems to also be predominant as the primary pattern of PMA as it travels alongside the MN and separates the distal end, diverging as it enters hand compartments [4,5,8,19,20]. In other studies, the PMA has also been described to travel alongside the median nerve more laterally as it descends into the carpal tunnel without ever splitting the terminal ends, which is different compared to the anterior-piercing type [2,3,6,21]. Likewise, studies have found similar characteristics with the medial traveling route described within this study [3,6,14].

Interestingly, the PMA has been known to stem from various main blood vessels, such as the ulnar artery, or come off various entry points of the anterior interosseous artery [3]. After branching off these vessels, the PMA then travels distally down the forearm between the proximal portions of the flexor digitorum superficialis and profundus, onward toward the hand compartment [3,17]. PMA then joins the vasculature of the superior palmar arch by completing the circuit with the ulnar artery [3,12,14]. In another instance, the PMA is also described to contribute to the blood supply of common digital arteries located distal to the bifurcation of the median nerve [3,4,6,10]. Within the described route and vasculature of the PMA, irritation or disruption of its blood flow has been reported to cause upper arm and hand pain symptoms commonly seen after upper arm operative procedures [1,3,7,16].

Limitations

This study also has some limitations that need to be taken into consideration. Retraction and ablation of the median nerve and surrounding muscles, along with fascia, have a high risk of accidental abruption of the PMA. As the PMA is naturally a fragile structure, transection is a foreseeable limitation. In addition, due to the preservation conditions of the cadaveric samples, size measurements of PMA diameter may vary in comparison to in vitro measures per imaging [5-8,17,19].

## Conclusions

The prevalence found in this study is consistently within the range of values portrayed in previous literature. The PMA has been reported in imaging studies to appear as an artery splitting or found within the peripheral proximity of the median nerve as it begins to travel into the carpal tunnel. The average diameter of the PMA is also within the ranges of previous literature in regard to data associated with open surgery.

Assessing the prevalence and orientation of the PMA in reference to the proximity of the carpal tunnel is crucial, as this vessel is commonly known to contribute to tunnel compression and post-surgical complications, including transient hand pain and motor dysfunction symptoms. The presence of the PMA should be assessed prior to surgical intervention to minimize carpal tunnel compression and postoperative complications. Further refined understanding of the PMA may also improve future non-surgical, pharmacological approaches and outcome management of both acute and chronic symptomatic episodes, including ischemia, vascular-related congestion, thrombosis risk, or neurovascular involvement contributing to pain and motor dysfunction.
